# Association of *ADRB2* rs1042713 with Obesity and Obesity-Related Phenotypes and Its Interaction with Dietary Fat in Modulating Glycaemic Indices in Malaysian Adults

**DOI:** 10.1155/2019/8718795

**Published:** 2019-03-17

**Authors:** Soma Roy Mitra, Pui Yee Tan, Farahnaz Amini

**Affiliations:** ^1^School of Biosciences, Faculty of Science, University of Nottingham Malaysia Campus, Semenyih, Malaysia; ^2^School of Healthy Aging, Medical Aesthetics & Regenerative Medicine, UCSI University, KL Campus, Kuala Lumpur, Malaysia

## Abstract

Gene-diet interaction studies have reported that individual variations in phenotypic traits may be due to variations in individual diet. Our study aimed to evaluate (i) the association of *ADRB2* rs1042713 with obesity and obesity-related metabolic parameters and (ii) the effect of dietary nutrients on these associations in Malaysian adults. *ADRB2* genotyping, dietary, physical activity, anthropometric, and biochemical data were collected from 79 obese and 99 nonobese individuals. Logistic regression revealed no association between *ADRB2* rs1042713 and obesity (*p*=0.725). However, the carriers of G allele (AG + GG genotypes) of rs1042713 were associated with increased odds of insulin resistance, 2.83 (CI = 1.04–7.70, adjusted *p*=0.042), in the dominant model, even after adjusting for potential confounders. Obese individuals carrying the G allele were associated with higher total cholesterol (*p*=0.011), LDL cholesterol levels (*p*=0.008), and total cholesterol/HDL cholesterol ratio (*p*=0.048), compared to the noncarriers (AA), even after adjusting for potential confounders. Irrespective of obesity, the carriers of GG genotype had significantly lower fasting glucose levels with low saturated fatty acid intake (<7.3% of TE/day) (4.92 ± 0.1 mmol/L vs 5.80 ± 0.3 mmol/L, *p*=0.011) and high intake of polyunsaturated fatty acid:saturated fatty acid ratio (≥0.8/day) (4.83 ± 0.1 mmol/L vs 5.93 ± 0.4 mmol/L, *p*=0.006). Moreover, the carriers of GG genotype with high polyunsaturated fatty acid intake (≥6% of TE/day) had significantly lower HOMA-IR (1.5 ± 0.3 vs 3.0 ± 0.7, *p*=0.026) and fasting insulin levels (6.8 ± 1.6 *µ*U/mL vs 11.4 ± 2.1 *µ*U/mL, *p*=0.036). These effects were not found in the noncarriers (AA). In conclusion, G allele carriers of *ADRB2* rs1042713 were associated with increased odds of insulin resistance. Obese individuals carrying G allele were compromised with higher blood lipid levels. Although it is premature to report gene-diet interaction on the regulation of glucose and insulin levels in Malaysians, we suggest that higher quantity of PUFA-rich food sources in regular diet may benefit overweight and obese Malaysian adults metabolically. Large-scale studies are required to replicate and confirm the current findings in the Malaysian population.

## 1. Background

Obesity and related chronic diseases have become the leading causes of morbidity and mortality worldwide. Long-term consumption of energy dense food coupled with sedentary lifestyle are the main contributors to the development of obesity and related complications [1]. Multiple genetic loci determined by genome-wide association studies (GWAS) have been found to be associated with increased susceptibility to obesity, diabetes, and dyslipidemia [2]. Earlier studies have reported on the adrenergic receptor system for its role in the stimulation of thermogenesis and in activating lipid mobilization from the fat stores. With respect to this, the beta-2 adrenoceptor gene is a candidate gene since it is the dominating lipolytic receptor in the human white adipose tissue [3]. It stimulates lipid mobilization through lipolysis in adipocytes and regulates body fat accumulation and energy expenditure [4].

A strong association between obesity and a single nucleotide polymorphism located at codon 16 substituting arginine for glycine (rs1042713/Arg16Gly) has been reported by Large et al. [5]. Masuo et al. reported that insulin-resistant subjects had higher frequencies of the G allele of rs1042713 [6]. Total body fat mass and blood pressure levels were higher in nonobese and nonhypertensive men with G allele in the Japanese population. The authors speculated that insulin resistance could, in part, be determined by the genetic variant of the beta-2 adrenoceptor gene and that polymorphism and higher plasma adrenaline could increase insulin resistance, adiposity, and high blood pressure in their subjects. Thus, the G allele could lead to heightened sympathetic nerve activity, insulin resistance, and higher blood pressure and adiposity in nonobese and nonhypertensive individuals. Other studies have adequately reported that insulin resistance was strongly associated with heightened sympathetic nerve activity [7]. In other words, adrenergic receptor defects lead to the sympathetic nervous system over activity that may play a role in the development of insulin resistance, hypertension, and obesity [8].

A study on the Swedish population reported that *ADRB2* rs1042713 was significantly associated with elevated central body fat, systolic blood pressure, serum leptin, and triglyceride levels but not with obesity [9]. Studies from Saudi Arabia reported significant association between *ADRB2* rs1042713 polymorphism and the development of insulin resistance, dyslipidemia, overweight, and obesity [10, 11]. However, findings from Asian populations (Japanese and Korean) reported negative association between obesity and *ADRB2* gene polymorphisms. Moreover, these studies did not investigate interaction of gene variants with dietary nutrients [12, 13].

Individual or population differences in the development of obesity-related metabolic diseases may result not only from genetic variation but may also be the modulatory effect of dietary nutrients on gene and gene variants [14]. In the Malaysian population, relatively little is known with respect to the interaction between dietary nutrients and *ADRB2* gene variations on obesity, insulin resistance, and glucose homeostasis. It is believed that early identification of the candidate gene variants and their interaction with diet may allow for the provision of good quality personalised dietary recommendation to achieve effective weight loss and reduction in metabolic risk factors [15]. Although a couple of intervention studies have reported lipid outcomes associated with *ADRB2* rs1042713, there is no such study done in Malaysian adults. Since there has been conflicting results with respect to *ADRB2* rs1042713 between Asian, Caucasian, and Arabic populations, the current study on the Malaysian population is valuable and will shed light and add to the existing evidence on gene variants and phenotypic outcomes and influence of diet on the latter. To the best of our knowledge, this is the first study in Malaysian adults that investigates the interaction between *ADRB2* rs1042713 and dietary nutrients on obesity-related metabolic traits. This study is nested in a broader study investigating the association of single nucleotide polymorphisms in genes that have widely been reported to influence obesity and obesity-related metabolic disorders in human individuals. In an earlier publication, we have reported that *FTO* rs9930506 may interact with dietary protein and Vitamin E intake and modulate hsCRP levels in our Malaysian participants [16]. The aim of the current study was to evaluate (i) the effect of *ADRB2* rs1042713 on obesity and obesity-related anthropometric and blood biochemical parameters and (ii) the influence of diet on the association between *ADRB2* rs1042713 and obesity phenotypes, in Malaysian adults.

## 2. Methods

### 2.1. Ethical Approval

This study was reviewed and approved by the University of Nottingham Malaysia Campus (UNMC) Science and Engineering Research Ethics Committee and was registered under Medical Research and Ethics Committee (MREC) of National Medical Research Registry (Research ID: 25110), Ministry of Health Malaysia (MOH). Written informed consent was obtained from all participants.

### 2.2. Study Design

This cross-sectional study was conducted from 2014–2017 on Malaysian adults aged between 18 and 74 years. The study investigated (i) the association between *ADRB2* rs1042713 with obesity and insulin resistance; (ii) the association between *ADRB2* rs1042713 and phenotypes in obese and nonobese individuals; and (iii) the interaction between *ADRB2* rs1042713 and dietary nutrients on phenotypic traits.

### 2.3. Participant Selection

Detailed information on the study design and methods can be found in our earlier publication [16]. Therefore, with respect to assessment of anthropometric parameters, dietary nutrients analysis, and physical activity level assessment, we refer the readers to our earlier publication [16].

### 2.4. Blood Collection and Biochemical Analysis

10–12 hour fasting blood was collected into vacutainer tubes containing fluoride oxalate for plasma glucose analysis and vacutainer tubes with clot activator and gel (Becton Dickinson, Oxford, United Kingdom) for serum lipid profile (total cholesterol, triglyceride, and HDL cholesterol levels), insulin, and high-sensitivity C-reactive protein (hsCRP) analysis. The analysis for above biochemical parameters were assessed using Abbott Architect CI8200 Automatic System following the manufacturer's instructions. Homeostatic model assessment to estimate insulin resistance (HOMA-IR) was calculated by multiplying fasting plasma glucose (mmol/L) by fasting serum insulin (*µ*U/ml) and divided by 22.5 [17]. Low-density lipoprotein (LDL) cholesterol was calculated using the Friedewald formula: LDL cholesterol = total cholesterol − ((triglyceride/5) + HDL cholesterol) [18]. This information has been reported in our earlier publication [16]. This study investigates *ADRB2* rs1042713 gene polymorphism in the same population with the aim to evaluate (i) the effect of *ADRB2* rs1042713 on obesity and obesity-related anthropometric and blood biochemical parameters and (ii) the influence of diet on the association between *ADRB2* rs1042713 and obesity phenotypes.

### 2.5. Genotyping of *ADRB2* rs1042713 Gene Polymorphism

Five milliliters of whole blood was drawn from an antecubital vein into vacutainer tubes (Becton, Dickinson and Co., Franklin Lakes, NJ) containing EDTA. The genomic DNA was extracted from leukocytes using the MasterPure DNA Purification kit (Lucigen Corporation Middleton, WI, USA) according to the manufacturer's instructions. DNA samples were stored at −20°C until use. A DNA fragment of 310 bp containing rs1042713 was amplified by using polymerase chain reaction (PCR) for the identification of *ADRB2* rs1042713 gene polymorphism with specific primers (forward primer: 5′-CCGCCGTGGGTCCGCC-3′ and reverse primer: 5′-CCATGACCAGATCAGCAC-3′) derived from an earlier study [19]. PCR was performed using 5 *µ*l of genomic DNA (∼1 ng/*µ*l), 0.2 *µ*M forward primer, and reverse primer with 5 *µ*l of Taq 5X Master Mix. Thermal cycling was performed as follows: initial denaturing at 95°C for 5 min; 35 cycles of denaturation at 94°C for 45 s, annealing at 64°C for 40 s and extension at 72°C for 45 sec, and then a final extension at 72°C for 5 min. The amplicons were verified by using electrophoresis on 2% agarose gel and visualized under ultraviolet illumination after staining by ethidium bromide. The verified amplicons were then sequenced by using BigDye® terminator v3.1 cycle sequencing kit chemistry.

### 2.6. Power and Sample Size Calculation

We performed power calculation using software QUANTO, Version 1.2.4, to find the minimum detectable effect for a given sample size. This calculation takes into account the type 1 error rate of 0.05, and the population prevalence of insulin resistance (using a cut off of HOMA-IR ≥ 1.7) of 45%, as reported in the present study. Given that the minor allele frequency (A) of *ADRB2* rs1042713 in our study was 0.49 with 57 insulin-resistant and 69 non-insulin-resistant participants, we had 68.2% power to detect an effect of 2.83 (odds ratio) (dominant model).

With regard to the gene-diet interaction, given that the mean of fasting glucose levels in our population was 5.2 mmol/L, with a SD of 2.0, environmental effect (differences of fasting glucose levels between high and low PUFA : SFA ratio) of −0.4 (5.0 mmol/L–5.4 mmol/L), genetic effect (differences of fasting glucose levels between the carriers of G allele of *ADRB2* rs1042713 and the noncarriers (A)) of 0.3 (5.4 mmol/L − 5.1 mmol/L), and interaction effect of −1.1 (4.83 mmol/L–5.93 mmol/L), a power of 61% for the gene-diet interaction was computed.

### 2.7. Statistical Analysis

Statistical analysis was performed using the statistical package for social sciences (IBM SPSS statistic, Chicago, IL, USA, version 22). Data were expressed as mean ± standard error (SE) or number (percentage). Log transformation was performed to transform nonnormally distributed data into normally distributed data. Independent *t*-test and chi-squared test were performed to assess the differences between the two genotype groups (AA vs AG + GG) on baseline continuous variables and categorical variables, respectively. Allele frequency was estimated by gene counting and chi-squared test was used to assess deviation from Hardy–Weinberg equilibrium (HWE) [20]. To study the effect of *ADRB2* rs1042713 on obesity, data were dichotomised into obese and nonobese groups (obesity was defined by BMI ≥ 27.5 kg/m^2^) [21]. The AA genotype of *ADRB2* rs1042713 was used as the reference group in both codominant and dominant models, whereas the combination of AG and GG genotypes was used as the reference group in recessive model. Odds ratios (ORs) with 95% confidence intervals (95% CIs) were estimated for each genotype by logistic regression to determine the odds of obesity associated with gene variants, after adjusting for covariates age, gender, physical activity status, smoking status, and alcohol consumption. Same analysis was performed to study the odds of *ADRB2* gene variants on insulin resistance. Data were dichotomised into (i) non-insulin-resistant and insulin-resistant groups (using a cutoff of HOMA-IR ≥ 1.7 [22]).

Differences in means between gene variants in anthropometric, blood biochemical, and dietary parameters in obese and nonobese groups were assessed by using one-way analysis of covariance (ANCOVA). Adjustment for covariates such as age, gender, physical activity status, smoking status, alcohol consumption, BMI, WC, fat mass, body fat percent, and total energy intake were applied where appropriate. The intake of macronutrients (energy-adjusted) was dichotomised into two groups based on the median intake of the population. A multivariate general linear model (GLM) was used to investigate the effect of the interaction between dietary macronutrients and *ADRB2* rs1042713 on obesity-related metabolic traits, after adjusting for potential confounders (age, gender, physical activity status, smoking status, alcohol consumption, BMI, and total energy intake). A statistical probability level of *p* < 0.05 (two-sided) was considered significant. No significant association was found between rs1042713 and protein and carbohydrate intake on obesity-related traits. Therefore, the results were not reported.

## 3. Result

### 3.1. Baseline Characteristics

In total, 178 Malaysian adults (female = 154; male = 24) were recruited for anthropometric measurement and genetic analysis. For biochemical and dietary analysis, 126 participants (female = 106, male = 20) were available. General characteristics of the study participants are reported in Table 1. The ages between the two groups (AA vs AG + GG) did not differ significantly (43.4 ± 1.8 y vs 41.1 ± 1.0 y; *p*=0.286). The gender distribution was not significantly different between the gene variants in the genotype groups (AA vs AG + GG), females (84.1% vs 87.3%), and males (15.9% vs 12.7%) (*p*=0.587). Ethnicity distribution was not significantly different between the two genotype groups (*p*=0.556). No significant difference was found in physical activity (*p*=0.256), smoking (*p*=0.606), and alcohol consumption (*p*=0.717) between the two genotype groups. No significant difference was found in height (*p*=0.907), body weight (*p*=0.505), and BMI (*p*=0.427) between the two genotype groups (*p* > 0.05). Due to the higher number of female participants, all data analyses were adjusted for by gender.

### 3.2. Allele Frequencies of the Gene Variants of *ADRB2* rs1042713

G allele was the most frequent variant in our study population (51.1%). The allele frequency for the minor allele of rs1042713 (A allele) was 0.49, which did not deviate from Hardy–Weinberg equilibrium as tested by the chi-squared test, *χ* ^2^ = 0.36 (*χ* ^2^ < 3.841) (Table 2).

### 3.3. Association between *ADRB2* rs1042713 and Obesity and Insulin Resistance

Logistic regression was performed to examine the independent effect of *ADRB2* rs1042713 on the odds of obesity and insulin resistance. We found no significant association between *ADRB2* rs1042713 and obesity (obesity as defined by BMI ≥ 27.5 kg/m^2^) under codominant (AG *p*=0.548 and GG, *p*=0.884), dominant (*p*=0.725), and recessive (*p*=0.538) models, after adjusting for covariates age, gender, physical activity status, smoking status, and alcohol consumption (Table 2).

However, our results revealed significant association between *ADRB2* rs1042713 and insulin resistance (using a cutoff of HOMA-IR ≥ 1.7) (Table 3). The carriers of GG genotype of rs1042713 had increased odds of insulin resistance, compared to AA genotype in both codominant and dominant models, 4.43 (CI = 1.31–15.0, adjusted *p*=0.016) and 2.83 (CI = 1.04–7.70, adjusted *p*=0.042), respectively, even after adjusting for covariates age, gender, BMI, physical activity status, smoking status, and alcohol consumption. No significant association was found in the recessive model (adjusted *p*=0.060).

### 3.4. Differences in Means between *ADRB2* rs1042713 Gene Variants in Anthropometric, Biochemical, and Dietary Parameters in Obese and Nonobese Groups

The age, anthropometric, biochemical, and dietary parameters of the study participants between gene variants in obese and nonobese groups have been reported in Table 4. In obese participants, we found that the carriers of G allele of rs1042713 had significantly higher total cholesterol (*p*=0.011), LDL cholesterol levels (*p*=0.008), and total cholesterol per HDL cholesterol ratio (*p*=0.048), compared to the noncarriers (AA), even after adjusting for covariates age, gender, BMI, WC, fat mass, body fat percent, physical activity status, smoking status, and alcohol consumption. Interestingly, such differences in blood biochemical parameters within genotypes were not observed in the nonobese group. With respect to dietary parameters, we found that the carriers of the G allele had significantly lower consumption of PUFA compared to the noncarriers (AA), even after adjusting for covariates (*p*=0.036). No significance association was found between *ADRB2* rs1042713 and others dietary parameters.

### 3.5. Differences between Blood Biochemical Parameters and Respective Diagnostic Cutoffs in Obese Individuals Carrying *ADRB2* rs1042713 G Allele

In obese individuals carrying the G allele of *ADRB2* rs1042713, HOMA-IR was significantly higher than the diagnostic cut off (2.8 ± 0.3) (Table 5). Although HDL cholesterol levels (1.5 ± 0.1 mmol/L) were positively, significantly higher than 1 mmol/L (diagnostic cutoff), total cholesterol (5.7 ± 0.1 mmol/L) and LDL cholesterol levels (3.6 ± 0.1 mmol/L) were significantly higher than the respective cutoffs, indicating metabolic risk (Table 5).

### 3.6. Interaction between Dietary Fat Intake and *ADRB2* rs1042713 on Fasting Glucose Levels, Insulin Levels, and HOMA-IR

**Figure 1:**
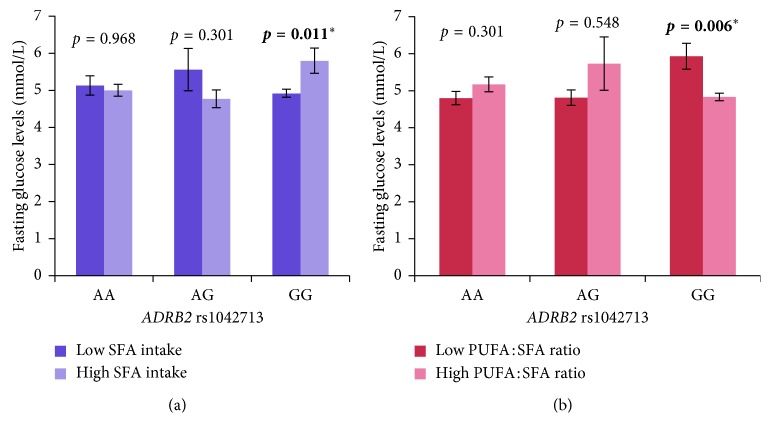
The effect of the interaction between *ADRB2* rs1042713 and (a) SFA intake and (b) PUFA : SFA ratio. SFA and PUFA : SFA ratios were dichotomised into two groups by using the median value of 7.3% of TE/day and 0.8/day (for all participants), respectively, for analysis. Gene-diet interaction was evaluated by using the multivariate general linear model after adjusting for age, gender, BMI, physical activity status, smoking status, alcohol consumption, and total energy intake. ^*∗*^ *p* < 0.05 was considered statistically significant. SFA: saturated fatty acid; PUFA: polyunsaturated fatty acid.

The multivariate general linear model was performed to investigate the effect of *ADRB2* rs1042713 and dietary macronutrients on phenotypic variations. In our study, our results revealed that, irrespective of obesity, the carriers of GG genotype of rs1042713 had significantly lower fasting glucose levels with low SFA intake (<7.3% of TE/day) (FBG: 4.92 ± 0.1 mmol/L vs 5.80 ± 0.3 mmol/L, *p*=0.011) (Figure 1(a)) and high PUFA : SFA ratio (≥0.8/day) (FBG: 4.83 ± 0.1 mmol/L vs 5.93 ± 0.4 mmol/L, *p*=0.006) (Figure 1(b)), even after adjusting for covariates age, gender, BMI, physical activity status, smoking status, alcohol consumption, and total energy intake.

**Figure 2:**
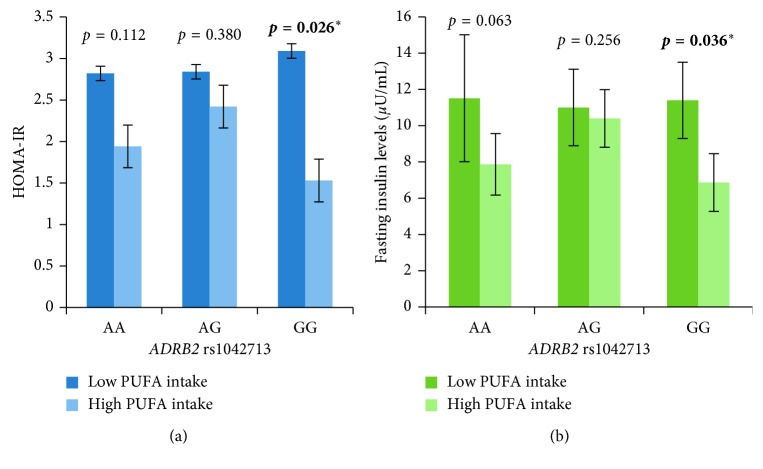
The effect of the interaction between *ADRB2* rs1042713 and PUFA intake on (a) HOMA-IR and (b) fasting insulin levels. PUFA intake was dichotomised into two groups by using the median value of 6% of TE/day (for all participants) for analysis. Gene-diet interaction was evaluated by using the multivariate general linear model after adjusting for age, gender, BMI, physical activity status, smoking status, alcohol consumption, and total energy intake. ^*∗*^ *p* < 0.05 was considered statistically significant. PUFA: polyunsaturated fatty acid; HOMA-IR: homeostatic model assessment-insulin resistance.

Moreover, the carriers of GG genotype of rs1042713 with high PUFA intake (≥6% of TE/day) had significantly lower HOMA-IR (1.5 ± 0.3 vs 3.0 ± 0.7, *p*=0.026) (Figure 2(a)) and fasting insulin levels (6.8 ± 1.6 *µ*U/mL vs 11.4 ± 2.1 *µ*U/mL, *p*=0.036) (Figure 2(b)) compared to low intake.

## 4. Discussion

Single-nucleotide polymorphism (SNP) in *ADRB2* rs1042713 causes alterations in the structural conformation of the receptor which eventually affect the function of *β*-adrenergic receptors (ADRB) [27]. This may influence the binding of catecholamines to the beta-2 adrenoceptors and hence alter lipolysis. A meta-analysis involving 18 published articles revealed that there was no association between rs1042713 and obesity [28]. Contrary to the latter, studies on the Saudi population reported significant association between *ADRB2* rs1042713 polymorphism and the development of obesity, as also with insulin resistance and dyslipidemia [10, 11]. However, findings from Asian populations (Japanese and Korean) reported negative association between obesity and *ADRB2* gene polymorphisms [12, 13]. Moreover, these studies did not investigate interaction of gene variants, phenotypes, and dietary nutrients. To date, there are no data reported on *ADRB2* rs1042713 in the Malaysian population. To the best of our knowledge, this is the first study that investigates the effect of *ADRB2* rs1042713 on obesity and obesity-related metabolic parameters and its interaction with dietary nutrients in Malaysian adults.

### 4.1. Association between *ADRB2* rs1042713 and Obesity and Insulin Resistance

In our study, we found no association between *ADRB2* rs1042713 and odds of obesity (obesity was defined as BMI ≥ 27.5 kg/m^2^). However, *ADRB2* rs1042713 was associated with insulin resistance (using a cutoff of HOMA-IR ≥ 1.7). We found that the carriers of G allele of rs1042713 had increased odds of insulin resistance compared to the noncarriers (AA), in the dominant model, even after adjusting for potential confounders. These findings suggest that variations in *ADRB2* rs1042713 may interfere with glucose homeostasis and cause insulin resistance. Prior et al. reported that *ADRB2* Arg16Gly–Gln27Glu haplotype was associated with glucose intolerance and insulin resistance in obese postmenopausal women [29]. A possible explanation for this observation could be the alteration in the structural conformation of the receptor, which may have enhanced sympathetic stimulation leading to increased lipolysis [8]. This overstimulation of *ADRB2* is found to be associated with the pathogenesis of insulin resistance as it inhibits the insulin-induced translocation of GLUT4 and reduces glucose uptake via the cAMP-dependent protein kinase A-dependent pathways [30].

We found that HOMA-IR in the obese individuals carrying G allele of *ADRB2* rs1042713 was above the diagnostic cutoff (1.7). It is now an established fact that higher levels of nonesterified fatty acids (NEFA) in the blood can induce preferential use of free fatty acids over glucose to generate ATP even in the presence of insulin in muscle and adipose tissue resulting in hyperglycaemia [31, 32]. Stimulation by noradrenaline of adipose tissue *ADRB2* increases the release of NEFA. In addition, free fatty acids (FFAs) can stimulate hepatic gluconeogenesis and alter pancreatic insulin release and subsequent metabolism in individuals with impaired glucose metabolism [33].

### 4.2. Association between *ADRB2* rs1042713 and Blood Lipid Levels

In the present study, we report that obese individuals carrying the G allele of rs1042713 had significantly higher total cholesterol, LDL cholesterol levels, and total cholesterol per HDL cholesterol ratio compared to the noncarriers (AA). Total cholesterol (5.7 ± 0.1 mmol/L) and LDL cholesterol levels (3.6 ± 0.1 mmol/L) in obese individuals carrying the G allele were above the diagnostic cutoffs (5.2 mmol/L and 2.6 mmol/L, respectively [26]). However, these differences in biochemical parameters were not observed in nonobese individuals. In our participants, excess FFAs in circulation in obese individuals carrying the gene variants of *ADRB2* may have driven dyslipidemia. Increased lipolysis due to the polymorphisms of *ADRB2* gene may have caused increased levels of NEFA induced hepatic production of VLDL and hence higher LDL levels in our participants [34].

### 4.3. Interaction between Dietary Fats and *ADRB2* rs1042713 on Glycaemic Indices

We report that the level of fasting glucose was modulated by the types of dietary fatty acids such as SFA and PUFA in the carriers of GG genotype of *ADRB2* rs1042713. The carriers of GG genotype of rs1042713 had significantly lower fasting glucose levels with intake of relatively higher PUFA:SFA ratio (≥0.8/day) (Figure 1(b)) and lower SFA intake (<7.3% of TE/day) (Figure 1(a)). Moreover, the carriers of GG genotype of rs1042713 had significantly lower fasting insulin levels and HOMA-IR when consuming higher PUFA intake (≥6% of TE/day).

These findings suggest that carriers of GG genotype of *ADRB2* rs1042713 consuming higher percentage of PUFA demonstrated better homeostatic control of fasting blood glucose and insulin sensitivity. A meta-analysis of 102 randomised controlled feeding trials with 4,200 subjects has reported that PUFA had the most beneficial effects in improving glycaemia, insulin resistance, and insulin secretion in comparison to dietary carbohydrate, SFA, and MUFA [35]. The anti-inflammatory properties of PUFA increase the production of adiponectin via PPARα activation and alleviate adipose tissue inflammation via GPR120 and resolvins/protectins, which favour insulin sensitivity. It also suppresses oxidative stress and pancreatic lipotoxicity, reduces toxicity of tissue free fatty acids, and increases membrane fluidity [36]. This body of evidence indicates that the composition of dietary fatty acid intake plays an important role in affecting glucose metabolism and insulin sensitivity.

As per Malaysian recommendations, 10% of total energy should come from SFA and 3 to 8% of total energy should come from PUFA [23]. On an average, dietary intake of our participants with respect to SFA and PUFA were within these ranges. However, individuals with G allele of rs1042713 may need to consume higher quantity of PUFA to combat diet-related noncommunicable diseases. Replacing isocaloric quantity of foods rich in SFA with PUFA may improve homeostatic control of blood glucose and enhance insulin sensitivity (HOMA-IR) in such individuals.

## 5. Limitations

A major limitation of our study is the small sample size. We acknowledge that the current study is exploratory and is underpowered to detect the gene-diet interaction between *ADRB2* rs1042713 and dietary nutrients on phenotypic and metabolic alterations in our population. However, with association and interaction analysis in the current study, we have generated a hypothesis. In future, large-scale studies are required to confirm such findings in the Malaysian population. In this study, we did not stratify our participants by gender for analysis due to the small sample size of male participants. To account for this, we have adjusted for gender statistically in all our data analysis to eliminate Type 1 error.

## 6. Conclusion

In conclusion, our study revealed that there was no association between *ADRB2* rs1042713 and obesity. However, *ADRB2* rs1042713 was associated with insulin resistance in Malaysian adults. The carriers of G allele of rs1042713 had increased odds of insulin resistance compared to noncarriers (AA). Obese individuals carrying the G allele of rs1042713 had significantly higher total cholesterol, LDL cholesterol levels, and total cholesterol per HDL cholesterol ratio compared to the noncarriers (AA). These differences were not observed in nonobese individuals. There is evidence from earlier studies that high PUFA intake is associated with favourable effects on glycaemia and insulin resistance. Over and above the latter, we found that higher PUFA intake was beneficial in individuals carrying the G allele with respect to glycaemic indices compared to the noncarriers. Although it is premature to report gene-diet interaction on the regulation of glucose and insulin levels in Malaysians, we suggest that higher quantity of PUFA-rich food sources in regular diet may benefit overweight and obese Malaysian adults metabolically. Large-scale studies are required to replicate and confirm the current findings in the Malaysian population.

## Figures and Tables

**Table 1:** General characteristics of the study participants; differences between *ADRB2* rs1042713 gene variants.

Genotype	*ADRB2* rs1042713 (dominant model)		
	AA (*n*=44)	AG + GG (*n*=134)	*p* value
Age (years)	43.4 ± 1.8	41.1 ± 1.0	0.286
*Gender*			
Female (*n*)	37 (84.1%)	117 (87.3%)	0.587
Male (*n*)	7 (15.9%)	17 (12.7%)	
*Ethnicity*			
Malays	20 (23.3%)	66 (46.7%)	0.556
Chinese	13 (31%)	29 (69%)	
Indians	11 (22%)	39 (78%)	
*Physical activity status*			
Physically active	41 (24%)	130 (76%)	0.256
Physically inactive	3 (42.9%)	4 (57.1%)	
*Smoking status*			
Never	44 (25%)	132 (75%)	0.606
Former	0	1 (100%)	
Current	0	2 (100%)	
*Alcohol consumption status*			
Never	44 (25.1%)	131 (74.9%)	0.717
Former	0	1 (100%)	
Current	0	1 (100%)	
Weight (kg)	65.7 ± 1.1	68.1 ± 1.6	0.505
Height (cm)	157.9 ± 1.1	157.8 ± 0.6	0.907
BMI (kg/m^2^)	26.3 ± 0.7	27.2 ± 0.5	0.427

^a^ *p* value based on the independent *t*-test. ^b^ *p* value based on the chi-squared test. *p* < 0.05 was considered as significant. Physically active was defined as accumulation of at least 150 minutes/week of moderate intensity activity (3–6 METs) or 60 minutes/week of vigorous physical activity (>6 METs) as defined by the Ministry of Health Malaysia [23].

**Table 2:** Genotype distribution and allele frequency of *ADRB2* rs1042713 in obese and nonobese groups.

Genotype	Overall *n*=178	*Χ* ^2^ (Hardy–Weinberg)	Obese BMI ≥ 27.5 *n*=79	Nonobese BMI < 27.5 *n*=99	Unadjusted OR (95% CI)	*p* value	Adjusted OR (95% CI)^*∗*^	*p* value^*∗*^
*Codominant model*								
AA	44 (24.7%)	0.36	19 (24.0%)	25 (25.2%)	1	—	1	—
AG	86 (48.3%)		41 (51.9%)	45 (45.5%)	1.20 (0.58–2.49)	0.627	1.26 (0.59–2.71)	0.548
GG	48 (27.0%)		19 (24.1%)	29 (29.3%)	0.86 (0.38–1.98)	0.726	0.94 (0.40–2.23)	0.884
*Dominant model*								
AA	44 (24.7%)		19 (24.0%)	25 (25.2%)	1	—	1	—
AG + GG	134 (75.3%)		60 (76.0%)	74 (74.8%)	1.07 (0.54–2.12)	0.853	1.14 (0.56–2.33)	0.725
*Recessive model*								
AA + AG	130 (73.0%)		60 (75.9%)	70 (70.7%)	1	—	1	—
GG	48 (27.0%)		19 (24.1%)	29 (29.3%)	0.76 (0.39–1.50)	0.434	0.80 (0.40–1.61)	0.538
*Allele frequency*								
A	174 (48.9%)		79 (50.0%)	95 (48.0%)				
G	182 (51.1%)		79 (50.0%)	103 (52.0%)				

The agreement of genotype frequencies with Hardy–Weinberg equilibrium was tested by using the chi-squared test, with *χ* ^2^ < 3.841 considered as no deviation from Hardy–Weinberg equilibrium. Logistic regression was conducted to determine the risk of obesity associated with gene variants. Odds ratios (ORs) with 95% confidence intervals (95% CIs) were estimated for each genotype. ^*∗*^Adjusted for age, gender, physical activity status, smoking status, and alcohol consumption. *p* < 0.05 was considered significant.

**Table 3:** Genotype distribution and allele frequency of *ADRB2* rs1042713 in insulin-resistant and non-insulin-resistant groups.

Genotype	HOMA < 1.7 *n*=69	HOMA ≥ 1.7 *n*=57	Unadjusted OR (95% CI)	*p* value	Adjusted OR (95% CI)^*∗*^	*p* value^*∗*^
*Codominant model*						
AA	21 (30.5%)	9 (15.8%)	1	—	1	—
AG	33 (47.8%)	30 (52.6%)	2.12 (0.84–5.35)	0.111	2.31 (0.81–6.61)	0.118
GG	15 (21.7%)	18 (31.6%)	2.80 (1.0–7.91)	0.052	**4.43 (1.31–15.0)**	**0.016**
*Dominant model*						
AA	21 (30.5%)	9 (15.8%)	1	—	1	—
AG + GG	48 (69.5%)	48 (84.2%)	2.33 (0.97–5.61)	0.058	**2.83 (1.04–7.70)**	**0.042**
*Recessive model*						
AA + AG	54 (78.3%)	39 (68.4%)	1	—	1	—
GG	15 (21.7%)	18 (31.6%)	1.66 (0.75–3.70)	0.213	2.46 (0.96–6.27)	0.060
*Allele frequency*						
A	75 (54.3%)	48 (42.1%)				
G	63 (45.7%)	66 (57.9%)				

Odds ratios (ORs) with 95% confidence intervals (95% CIs) were estimated for each genotype by logistic regression to determine the risk of insulin resistance associated with *ADRB2* rs1042713. *p* ≤ 0.05 was considered as significant. ^*∗*^Adjusted for age, BMI, gender, physical activity status, smoking status, and alcohol consumption. Non-insulin-resistant and insulin-resistant groups were dichotomised using a cutoff of HOMA-IR ≥ 1.7 [22].

**Table 4:** Differences in means (±SE) between *ADRB2* rs1042713 gene variants in anthropometric, biochemical, and dietary parameters in obese and nonobese groups.

	*ADRB2* rs1042713 (dominant model)					
	Obese (*n*=79)			Nonobese (*n*=99)		
*General characteristics*	AA (*n*=19)	AG + GG (*n*=60)	*p* value	AA (*n*=25)	AG + GG (*n*=74)	*p* value
Age (years)	**47.8** **±** **2.0**	**42.8** **±** **1.5**	**0.038** ^*∗*^	40.0 ± 2.7	39.7 ± 1.3	0.958
^1^Weight (kg)	76.0 ± 2.0	81.2 ± 2.3	0.097	57.9 ± 1.9	57.4 ± 1.0	0.957
^1^Height (cm)	157.7 ± 1.7	157.7 ± 1.0	0.688	158.1 ± 1.5	157.9 ± 0.8	0.871
^1^BMI (kg/m^2^)	30.5 ± 0.5	32.4 ± 0.6	0.086	23.0 ± 0.6	23.0 ± 0.3	0.936
^1^WC (cm)	97.6 ± 2.2	99.4 ± 1.6	0.173	68.6 ± 3.7	68.6 ± 2.0	0.899
^1^WHR	0.90 ± 0.01	0.93 ± 0.01	0.117	0.87 ± 0.01	0.87 ± 0.01	0.728
^1^Muscle mass (kg)	24.0 ± 0.9	24.4 ± 0.7	0.525	20.9 ± 1.0	20.9 ± 0.6	0.941
^1^Fat mass (kg)	32.3 ± 1.2	36.6 ± 1.4	0.065	19.2 ± 1.0	19.5 ± 0.6	0.887
^1^Fat-free mass (kg)	43.8 ± 1.6	44.6 ± 1.2	0.475	38.6 ± 1.6	37.9 ± 0.7	0.678
^1^Percent body fat (%)	42.5 ± 1.2	44.8 ± 0.7	0.110	33.3 ± 1.3	33.8 ± 0.8	0.683
^1^Systolic BP (mmHg)	124.0 ± 3.6	124.0 ± 2.0	0.712	122.6 ± 5.0	119.6 ± 2.3	0.587
^1^Diastolic BP (mmHg)	81.0 ± 2.3	81.5 ± 1.3	0.535	81.9 ± 2.7	79.2 ± 1.8	0.588
^1^Pulse rate (bpm)	75.7 ± 2.5	76.1 ± 1.3	0.948	75.1 ± 4.5	77.9 ± 2.0	0.556

*Blood biochemical parameters*	AA (*n*=19)	AG + GG (*n*=60)	*p* value	AA (*n*=11)	AG + GG (*n*=36)	*p* value
^2^Fasting glucose (mmol/L)	5.1 ± 0.2	5.3 ± 0.3	0.509	5.0 ± 0.3	5.2 ± 0.3	0.561
^2^Fasting insulin (uU/mL)	7.74 ± 1.6	11.2 ± 1.3	0.209	11.7 ± 3.7	7.5 ± 1.4	0.361
^2^HOMA-IR	1.9 ± 0.5	2.8 ± 0.3	0.176	2.9 ± 1.0	2.2 ± 0.7	0.494
^2^Total cholesterol (mmol/L)	**5.0** **±** **0.2**	**5.7** **±** **0.1**	**0.011** ^*∗*^	5.4 ± 0.3	5.6 ± 0.2	0.942
^2^Triglyceride (mmol/L)	1.2 ± 0.2	1.4 ± 0.1	0.083	1.7 ± 0.4	1.4 ± 0.1	0.983
^2^HDL cholesterol (mmol/L)	1.6 ± 0.1	1.5 ± 0.1	0.791	1.5 ± 0.1	1.6 ± 0.1	0.636
^2^LDL cholesterol (mmol/L)	**2.9** **±** **0.2**	**3.6** **±** **0.1**	**0.008** ^*∗*^	3.3 ± 0.4	3.3 ± 0.2	0.650
^2^Total cholesterol/HDL cholesterol	**3.4** **±** **0.2**	**3.9** **±** **0.1**	**0.048** ^*∗*^	3.7 ± 0.3	3.7 ± 0.2	0.650
^2^hsCRP (mg/L)	5.2 ± 1.5	8.8 ± 1.4	0.059	2.2 ± 0.5	2.9 ± 0.5	0.750

*Dietary parameters*	AA (*n*=19)	AG + GG (*n*=60)	*p* value	AA (*n*=11)	AG + GG (*n*=36)	*p* value
^3^Total energy intake (kcal)	2145.5 ± 108.0	2065.6 ± 41.7	0.766	1897.5 ± 53.6	1867.5 ± 47.4	0.836
^4^Actual total carbohydrate intake (g)	272.3 ± 20.7	259.4 ± 7.2	0.967	237.8 ± 13.1	234.0 ± 11.0	0.625
^4^Actual total protein intake (g)	75.1 ± 5.3	77.1 ± 3.1	0.982	72.8 ± 5.3	70.0 ± 3.2	0.276
^4^Actual total dietary fat intake (g)	90.2 ± 5.5	84.7 ± 3.1	0.810	77.1 ± 4.1	77.7 ± 2.6	0.817
^4^Percentage energy from carbohydrate (%)	46.9 ± 1.8	46.9 ± 1.2	0.995	46.8 ± 2.1	47.7 ± 1.3	0.520
^4^Percentage energy from protein (%)	13.8 ± 0.6	14.9 ± 0.5	0.799	15.5 ± 1.2	14.4 ± 0.7	0.181
^4^Percentage energy from dietary fat (%)	37.9 ± 1.9	36.4 ± 1.0	0.744	36.4 ± 1.5	36.8 ± 1.1	0.968
^4^SFA (%TE)	7.0 ± 1.0	7.6 ± 0.5	0.514	7.0 ± 0.8	8.7 ± 0.6	0.307
^4^MUFA (%TE)	11.8 ± 1.1	10.3 ± 0.6	0.339	11.8 ± 1.1	12.1 ± 0.6	0.895
^4^PUFA (%TE)	**8.0** **±** **0.8**	**5.6** **±** **0.4**	**0.036** ^*∗*^	6.5 ± 1.1	7.0 ± 0.6	0.523
^4^Trans fat (%TE)	0.1 ± 0.01	0.1 ± 0.04	0.109	0.1 ± 0.03	0.1 ± 0.02	0.497
^4^Dietary cholesterol (%TE)	88.8 ± 14.0	108.3 ± 10.5	0.987	111.4 ± 22.9	109.7 ± 16.1	0.471

One-way analysis of covariance was performed to determine the differences between means between gene variants in anthropometric, biochemical, and dietary parameters in obese and nonobese participants, after adjusting for covariates in different model ^1^age, gender, physical activity status, smoking status, and alcohol consumption; ^2^model^1^ + BMI, WC, body fat mass, and body fat percent; ^3^model^1^ + BMI; and ^4^model^3^ + total energy intake. ^*∗*^ *p* < 0.05 was considered significant. HOMA-IR: homeostatic model assessment-insulin resistance; HDL: high-density lipoprotein; LDL: low-density lipoprotein; hsCRP: high-sensitivity C-reactive protein; SFA: saturated fatty acid; MUFA: monounsaturated fatty acid; PUFA: polyunsaturated fatty acid; % TE: percentage of total energy intake.

**Table 5:** Differences between blood biochemical parameters and respective diagnostic cutoffs in obese individuals carrying *ADRB2* rs1042713 G allele.

Blood biochemical parameters	Mean (±SE)	Diagnostic cutoff	*p* value
Fasting glucose (mmol/L)	5.3 ± 0.3	5.6 [24]	0.327
Fasting insulin (*µ*U/mL)	11.2 ± 1.3	10.6 [25]	0.330
HOMA-IR	**2.8** **±** **0.3**	**1.7** [22]	**0.003** ^*∗*^
Total cholesterol (mmol/L)	**5.7** **±** **0.1**	**5.2** [26]	**0.001** ^*∗*^
Triglyceride (mmol/L)	**1.4** **±** **0.1**	**1.7** [24]	**<0.001** ^*∗*^
HDL cholesterol (mmol/L)	**1.5** **±** **0.1**	**1.0** [24]	**<0.001** ^*∗*^
LDL cholesterol (mmol/L)	**3.6** **±** **0.1**	**2.6** [26]	**<0.001** ^*∗*^
Total cholesterol/HDL cholesterol	3.9 ± 0.1	—	—

The one-sample *t*-test was performed to assess the difference between blood biochemical parameters and respective diagnostic cutoffs ^*∗*^ *p* < 0.05 was considered significant. HOMA-IR: homeostatic model assessment-insulin resistance; HDL: high-density lipoprotein; LDL, low-density lipoprotein.

## Data Availability

The datasets generated and/or analysed during the present study are not publicly available, since ethical approval and participants' consent do not allow public sharing of data, but are available from the corresponding author upon reasonable request.

## Abbreviations

*ADRB2*: Beta-2 adrenergic receptor
AHA: American Heart Association
ANCOVA: One-way analysis of covariance
Arg: Arginine
BMI: Body mass index
BMR: Basal metabolic rate
cAMP: Cyclic adenosine monophosphate
CI: Confidence interval
DNA: Deoxyribonucleic acid
DSM-BIA: Direct segmental multifrequency-bioelectrical impedance analysis method
EDTA: Ethylenediaminetetraacetic acid
FFAs: Free fatty acids
GLM: General linear model
Gln: Glutamine
Glu: Glutamic acid
GLUT4: Glucose transporter type 4
Gly: Glycine
GPR120: G-protein coupled receptor 120
GWAS: Genome-wide association studies
HDL: High-density lipoproteins
HOMA: Homeostatic model assessment
HOMA-IR: Homeostatic model assessment-insulin resistance
hsCRP: High-sensitivity C-reactive protein
HWE: Handy–Weinberg equilibrium
LDL: Low-density lipoproteins
MET: Metabolic equivalent
MOH: Ministry of health Malaysia
MREC: Medical Research and Ethics Committee
MUFA: Monounsaturated fatty acid
NEFA: Nonesterified fatty acids
OR: Odds ratio
PCR: Polymerase chain reaction
PPAR*α*: Peroxisome proliferator-activated receptor alpha
PUFA: Polyunsaturated fatty acid
SFA: Saturated fatty acid
SE: Standard error of mean
SNPs: Single-nucleotide polymorphisms
TE: Total energy intake
UCP-1: Uncoupling protein 1
UCSI: University College Sedaya International
UNMC: University of Nottingham Malaysia Campus
VLDL: Very-low-density lipoproteins
vs: versus
WC: Waist circumference
WHO: World Health Organization
WHR: Waist hip ratio
*χ*^2^: Chi-square test.

## Supplementary Materials

Supplementary Materials Supplementary Table 1: physical activity and lifestyle of study participants.
