# Supplementary material for: Association of ADRB2 rs1042713 with Obesity and Obesity-Related Phenotypes and Its Interaction with Dietary Fat in Modulating Glycaemic Indices in Malaysian Adults
Source: J Nutr Metab. 2019 Mar 17;2019:8718795. doi: 10.1155/2019/8718795 (PMC6441509; doi:10.1155/2019/8718795)
Supplement: Supplementary Materials — Supplementary Table 1: physical activity and lifestyle of study participants. [file 8718795.f1.docx]

**Supplementary table 1: Physical activity and lifestyle of study participants**

Title of study- Association of *ADRB2* rs1042713 with obesity and obesity-related phenotypes and its interaction with dietary fat in modulating glycaemic indices in Malaysian adults

|  | **Total (n=178)** |
| --- | --- |
| **Physical activity status** |  |
| **Light (<3 METs)** | 154 (86.5%) |
| **Moderate (3-6 METs)** | 19 (10.7%) |
| **Vigorous (>6 METs)** | 5 (2.8%) |
| Result: 4% (n=7) of the study participants were considered as physically active* and 96% (n=171) were physically inactive. | |
| **Smoking status** |  |
| **Never** | 175 (98.3%) |
| **Former** | 1 (0.6%) |
| **Current** | 2 (1.1%) |
| **Alcohol consumption** |  |
| **Never** | 176 (98.8%) |
| **Former** | 1 (0.6%) |
| **Current** | 1 (0.6%) |

Note: *‘Physically active’ was defined as accumulation of at least 150 minutes/week of moderate intensity activity (3-6 METs) or 60 minutes/week of vigorous physical activity (>6 METs). This analysis was used to adjust for physical activity in the analysis of tables (2, 3, 4a and 4b) and figures (1a, 1b, 2a and 2b).
